# Supplementary material for: A Functional K+ Channel from Tetraselmis Virus 1, a Member of the Mimiviridae
Source: Viruses. 2020 Sep 29;12(10):1107. doi: 10.3390/v12101107 (PMC7650704; doi:10.3390/v12101107)
Supplement: Supplementary file 1 [file viruses-12-01107-s001.zip › viruses-941227-supplementary.pdf]

Supplemental Table 1. Virus names and abbreviations

Virus	Abbreviation
Tetraselmis virus 1	TetV-1
Chrysochromulina sp. virus SA1	CsV-KB1
Florenciella sp. virus SA2	FloV-SA2
Rhizochromulina sp. virus SA1	RhiV-SA1
Paramecium bursaria chlorella virus 1	PBCV-1
Only Syngen chlorella virus NE5	OSyNE5
Ectocarpus siliculosus virus-1	EsV1
Chlorella Pbi virus MT325	MT325
Acanthocystis turfacea chlorella virus-1	ATCV-1
Bathycoccus sp. RCC1105	BpV1
Micromonas sp. RCC1109 virus	MpV1
Ostreococcus tauri virus RT-2011	OtV6
Dishui Lake phycodnavirus 1	DSLPV1
Yellow Lake phycodnavirus	YLPV2
Aureococcus anophagefferens virus-1	AaV1
Organic Lake phycodnavirus-2	OLPV2
Mycobacterium phage Myrna	MpMyrna
Mycobacterium phage Phabba	MpPhabba
Acinetobacter phage vB_AbaM_ME3	AP
Lactobacillus phage PLE	LpPLE2
Vibrio phage 1.084.O._10N.261.49.F5	Vp2
Vibrio phage 2.275.O._10N.286.54.E11	Vp3
Vibrio phage 1.081.O._10N.286.52.C2	Vp1
Vibrio phage phi-pp2	Vpphipp2
Vibrio phage VH7D	VpVh7
